# Genomic Variant in *IL-37* Confers A Significant Risk of Coronary Artery Disease

**DOI:** 10.1038/srep42175

**Published:** 2017-02-09

**Authors:** Dan Yin, Duraid Hamied Naji, Yunlong Xia, Sisi Li, Ying Bai, Guiqing Jiang, Yuanyuan Zhao, Xiaojing Wang, Yufeng Huang, Shanshan Chen, Jingjing Fa, Chengcheng Tan, Mengchen Zhou, Yingchao Zhou, Longfei Wang, Ying Liu, Feifei Chen, Jingqiu Liu, Qiuyun Chen, Xin Tu, Chengqi Xu, Qing K. Wang

**Affiliations:** 1 Key Laboratory of Molecular Biophysics of the Ministry of Education, College of Life Science and Technology, Center for Human Genome Research, Cardio-X Institute, Huazhong University of Science and Technology, Wuhan, P. R. China; 2 BGI-Wuhan, Wuhan 430075, China; BGI-Shenzhen, Shenzhen 518083, China; 3 Cardiovascualr Hospital, the First Affiliated Hospital of Dalian Medical University, Dalian, Wuhan, P. R. China; 4 Center of Prenatal Diagnosis, the First Affiliated Hospital of Zhengzhou University, Zhengzhou, Hunan, P. R. China; 5 Key Laboratory for Molecular Diagnosis of Hubei Province, The Central Hospital of Wuhan, Tongji Medical College, Huazhong University of Science and Technology, Wuhan, Hubei Province, P. R. China; 6 Center for Cardiovascular Genetics, Department of Molecular Cardiology, Lerner Research Institute, Department of Cardiovascular Medicine, Cleveland Clinic, Cleveland, Ohio, USA; 7 Department of Molecular Medicine, Department of Genetics and Genome Science, Case Western Reserve University, Cleveland, OH, USA

## Abstract

The interleukin 1 family plays an important role in the immune and inflammatory responses. Coronary artery disease (CAD) is a chronic inflammatory disease. However, the genetic association between *IL-37*, the seventh member of the IL-1 family, and CAD is unknown. Here we show that a single nucleotide polymorphism in the *IL-37* gene (rs3811047) confers a significant risk of CAD. We have performed an association analysis between rs3811047 and CAD in two independent populations with 2,501 patients and 3,116 controls from China. Quantitative RT-PCR analysis has been performed to determine if the *IL-37* expression level is influenced by rs3811047. We show that the minor allele A of rs3811047 is significantly associated with CAD in two independent populations under a recessive model (*P*_*adj*_ = 5.51 × 10^−3^/OR = 1.56 in the GeneID Northernern population and *P*_*adj*_ = 1.23 × 10^−3^/OR = 1.45 in the GeneID Central population). The association became more significant in the combined population (*P*_*adj*_ = 9.70 × 10^−6^/OR = 1.47). Moreover, the association remains significant in a CAD case control population matched for age and sex. Allele A of rs3811047 shows significant association with a decreased mRNA expression level of *IL-37* (n = 168, *P* = 3.78 × 10^−4^). These data suggest that *IL37* is a new susceptibility gene for CAD, which provides a potential target for the prevention and treatment of CAD.

Coronary artery disease (CAD) is the leading cause of morbidity and mortality in the world^1^,^2^. Epidemiological and family studies revealed that CAD has a strong genetic component^3^. The heritability of CAD was shown to be from 40% to 60%^4^. Understanding the genetic risk factors of CAD or atherosclerosis (the cause of CAD)^5^ may provide important information on the biological pathways of CAD pathogenesis.

Recently, large-scale genome-wide association studies (GWAS) have identified more than 50 risk loci for CAD and provided new information to the biological pathways that were not associated with traditional risk factors of CAD before^6^,^7^,^8^,^9^,^10^,^11^,^12^. Although genome-wide approaches have provided novel insights into the genetic basis of CAD etiology, the GWAS risk loci have the modest effects and explain approximately 10% of CAD heritability^9^. Furthermore, GWAS may miss some specific candidate genetic variants^13^. Therefore, the genetic architecture of CAD remains to be further defined.

Cytokines and chronic inflammation play an important role in the pathogenesis of atherosclerosis and CAD^14^. Recent studies have found that many variants of genes encoding cytokines, such as IL-6, IL-10, IL-16, IL-17A, IL-18 and IL-33^15^,^16^,^17^,^18^,^19^,^20^, are genetically associated with atherosclerosis and CAD in humans. Evidence from animal studies has demonstrated that many cytokines regulated by interleukins participate in the pathological inflammatory processes involved in atherosclerosis^21^.

Interleukin-37 (IL-37/IL-1F7) is a member of the IL-1 cytokine family^22^, which broadly modulates inflammatory and immune responses^23^. IL-37 has a caspase-1 site and can be processed by caspase-1^24^. After caspase-1 processing, IL-37 is translocated to the nucleus and reduces the production of pro-inflammatory cytokines in murine RAW cells following LPS stimulation^25^. In 2010, Nold *et al*. observed that IL-37 was an anti-inflammatory cytokine, affected a broad spectrum of pro-inflammatory cytokines, and suppressed immune responses^26^. Subsequently, McNamee *et al*. observed that IL-37 played a protective and anti-inflammatory role by decreasing recruitment of leukocytes into the inflamed tissue. Furthermore, Ge *et al*. reported that SNP rs3811047, a functional tagSNP of *IL-37*, was associated with ankylosing spondylitis, which is an idiopathic inflammatory disease affecting the axial and/or peripheral skeleton with an increased risk of atherosclerosis and cardiovascular mortality and morbidity^27^,^28^. Based on these data, we hypothesized that *IL-37* may play a role in the pathogenesis of CAD and the functional tagSNP of *IL-37*, rs3811047, may be genetically associated with CAD. In the present study, we genotyped SNP rs3811047 in *IL-37* in two independent case control populations of CAD, and performed an association study to test whether the genetic variant in *IL37* confers a risk to CAD.

## Results

### Characteristics of study populations and power analysis

The demographic and clinical characteristics of two independent CAD populations used for case control analysis, including the GeneID Northern population and the GeneID Central population, are shown in Table 1. The GeneID Northern population included 1,038 CAD patients and 1,076 controls. The mean age was 62.01 ± 12.70 in cases and 50.52 ± 16.71 in controls. The proportion of males was 65.51% in cases and 69.33% in controls. The GeneID Central population enrolled 1,463 cases and 2,040 controls. The mean age was 64.21 ± 12.44 in cases and 49.02 ± 13.88 in controls, respectively. The proportion of males was 65.21% and 61.76% in cases and controls, respectively. The percentage of males in cases was higher than in controls because the male gender is a well-known risk factor for the development of CAD. The average age of the control populations is younger than that in the case populations because most controls are study subjects who had free physical examinations offered to active working individuals by their respective working institutions. The differences of age and sex between cases and controls were adjusted in later statistical analysis. To further reduce the confounding of age and sex, we generated a case control population by randomly matching each individual case to a control based on age and sex. The data on demographic and clinical characteristics of the matched case control population are shown in Table 1.

Under the population parameter setting of the effect size or odds ratio (OR) of 1.2 for CAD^29^, and the minor allele frequency of 0.209 for rs3811047 (HapMap CHB data sets), our samples provide a statistical power of 70% in the Northern population, and 88% in the Central population to detect an association between rs3811047 and CAD with a type I error of 0.05. The combined population has 2,501 cases and 3,116 controls and can provide a statistical power of 98%. The matched case control population has a power of 83%. Therefore, our GeneID samples are sufficiently large to test the association between SNP rs3811047 and CAD.

### Significant association between SNP rs3811047 in *IL37* and CAD in two independent Chinese populations

The genotyping data for SNP rs3811047 showed no deviation from the Hardy-Weinberg equilibrium in the control populations (*P* > 0.05). In the GeneID Northern population, the minor allele A of rs381047 confers a significant risk of CAD (*P*_*obs*_ = 6.72 × 10^−4^, OR = 2.45) under a recessive model (Table 2). After adjustment for covariates of age, sex, hypertension, diabetes, and lipid concentrations (TG, Tch, LDL-c and HDL-c), the significant association remained (*P*_*adj*_ = 5.51 × 10^−3^, OR = 1.56).

The association between SNP rs3811047 and CAD is the first time finding, therefore, we need to replicate the finding in another independent population. The replication study using the GeneID Central population showed that SNP rs3811047 was a significant risk factor for CAD (*P*_*obs*_ = 4.72 × 10^−4^, OR = 1.83; *P*_*adj*_ = 1.23 × 10^−3^, OR = 1.45) (Table 2) in the GeneID Central populaion, also under the recessive model (Table 2). Therefore, the association between rs3811047 and CAD was independently confirmed in the replication population.

Combination of the two independent populations together resulted in a larger population with 2,501 cases and 3,116 controls. The association between SNP rs3811047 and CAD became more significant in the combined population. Minor allele A of SNP rs3811047 showed a much more significant risk of CAD (*P*_*adj*_ = 9.70 × 10^−6^ with an OR of 1.47) under the recessive model (Table 2).

We also analyzed allelic association between SNP rs3811047 and CAD in the combined population (Table 3). A significant allelic association was found between rs3811047 and CAD (*P*_*obs*_ = 3.20 × 10^−4^, OR = 1.19). After adjustment for covariats of age, sex, hypertension, diabetes, and lipid concentrations (TG, Tch, LDL-c and HDL-c), the significant association remained (*P*_*adj*_ = 0.01, OR = 1.16) (Table 3).

The significant association between SNP rs3811047 and CAD remained significant after adjustment for age and sex, suggesting that the differences of age and the percentage of males between cases and controls did not affect the conclusion of our case control analysis. To further minimize the confounding of age and sex, we generated a case control population with each case randomly matched to a control by exact matching or one-by-one matching. Statistical analysis was then carried out to further test whether *IL-37* SNP rs3811047 is still significantly associated with CAD. As shown in Table 4, the minor allele A of rs3811047 conferred a significant risk of CAD in the matched case control population under a recessive model (*P*_*obs*_ = 4.57 × 10^−9^, OR = 2.82) and under an additive model (*P*_*obs*_ = 3.66 × 10^−8^). The association remained significant after adjustment of other covariates, including hypertension, diabetes, and lipid concentrations (TG, Tch, LDL-c and HDL-c) (*P*_*adj*_ = 2.23 × 10^−4^, OR = 1.99 under a recessive model; *P*_*adj*_ = 1.02 × 10^−3^, OR = 1.45 under an additive model) (Table 4). Allelic association analysis also showed that the minor allele A of SNP rs3811047 conferred a significant risk of CAD in the matched case control population before (*P*_*obs*_ = 1.60 × 10^−5^, OR = 1.31) and after adjustment of hypertension, diabetes, and lipid concentrations (TG, Tch, LDL-c and HDL-c) (*P*_*adj*_ = 6.43 × 10^−3^, OR = 1.26) (Table 5). We also analyzed the association between rs3811047 and CAD in a male only population and in a female only population, and significant association was identified in both populations (Table 5).

### Real time RT-PCR analysis idenfified significant association between SNP rs3811047 and the expression level of *IL-37* mRNA

**Figure 1:**
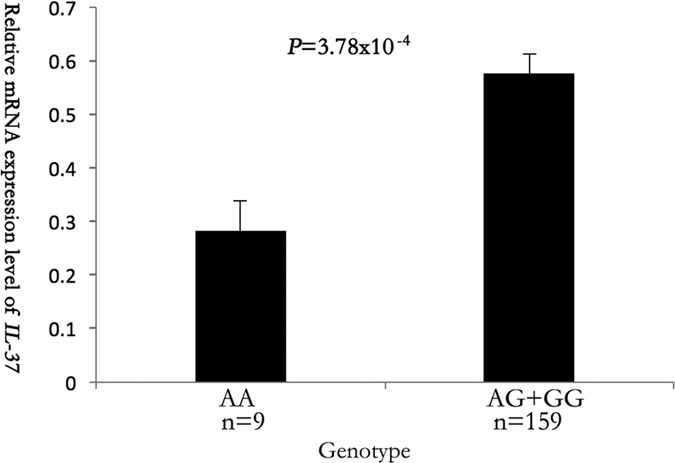
Assessment of the relationship between *IL-37* SNP rs3811047 and the expression level of *IL-37* mRNA by real time RT-PCR analysis. Total RNA samples were isolated from 168 blood samples (lymphocytes), converted into cDNA, and used for real time PCR analysis. Genomic DNA samples were isolated from the 168 study subjects and genotyped for SNP rs3811047 by HRM analysis. A linear regression was used to compare the differences for the mean RQ values between different genotypes (AA and AG + GG) of SNP rs3811047.

We carried out real time RT-PCR to analyze whether the expression level of *IL-37* is associated with the genotype of SNP rs3811047 using blood samples from 168 study subjects. The results showed that the expression level of the *IL-37* mRNA was significantly different among different genotypes under a recessive genetic model (*P* = 3.78 × 10^−4^) (Fig. 1). The expression level of the *IL-37* mRNA was significantly lower in carriers with the AA genotype than carriers with GG and GA genotypes (Fig. 1). Together, these results suggest that the minor allele A of SNP rs3811047 is significantly associated with a decreased expression of *IL-37*.

## Discussion

In the present study, we genotyped SNP rs3811047 in the *IL-37* gene in two independent case control populations of CAD, and performed an association study to test whether the genetic variant in *IL37* confers risk of CAD. Here we provide genetic evidence that minor allele A of rs3811047 in the *IL-37* gene was significantly associated with the risk of CAD in two independent case control populations (Tables 2 and 3). The association between SNP rs3811047 and CAD became even more significant in the combined population (Tables 2 and 3). The association between SNP rs3811047 and CAD remained significant after adjustment of covariates of age, sex, hypertension, diabetes, triglyceride, total cholesterol, LDL-cholesterol and HDL-cholesterol levels (Tables 2 and 3). Significant allelic and genotypic association between SNP rs3811047 and CAD was also identified in a case control population matched by age and sex (Tables 4 and 5). The significant allelic association remained in the separated male population and the female population (Table 5). These data suggest that SNP rs3811047 in *IL-37* is a risk factor of CAD independent from age, gender, hypertension, diabetes, and lipid levels. Moreover, we found that the minor allele A of SNP rs3811047 was associated with a decreased expression level of the *IL-37* mRNA. These results suggest that IL-37 is a susceptibility gene for CAD. This is the first study that establishes the significant association between an *IL-37* variant and CAD. Our studies used cases and controls from the GeneID Chinese Han population. We hope that the significant association between an *IL-37* variant and CAD can be replicated in other Chinese populations and even in other ethnic populations. In addition, future studies can also analyze whether the *IL-37* variant is also associated with the severity of CAD and other diseases such as ischemic stroke associated with inflammation.

SNP rs3811047 was first associated with human leukocyte antigen-B27 positive ankylosing spondylitis in the Chinese population^30^. The previous study observed that there was an interaction between *IL-37* gene and alcohol drinking in ankylosing spondylitis patients in a case-only study^31^. Ankylosing spondylitis is one of the most common chronic inflammatory autoimmune diseases affecting the axial and/or peripheral skeleton with an estimated prevalence of 0.1–0.9%^32^. Chronic inflammation and cytokines play important roles in the pathogenesis of atherosclerosis and CAD. There is evidence that ankylosing spondylitis patients have a higher risk of mortality and morbidity compared to the general population and also a higher rate of cardiovascular death^28^. Several studies also showed that cardiovascular diseases are more common in patients with ankylosing spondylitis^33^,^34^. These findings suggested that a common mechanism, such as chronic inflammation, may be shared between CAD and ankylosing spondylitis. We hypothesized that the genetic risk factors of ankylosing spondylitis may also play a role in the pathogenesis of atherosclerotic CAD. In the present study, for the first time, we show that SNP rs3811047 in *IL-37* is indeed a risk factor for CAD.

IL-37 is the seventh member of the IL-1 family, and considered as an anti-inflammatory cytokine which mainly inhibits the expression, production and function of other pro-inflammatory cytokines^35^. IL-37 is normally expressed at a low level in peripheral blood monocytes, but its expression is rapidly up-regulated in monocytes as well as dendritic cells under an inflammatory context^26^. This leads to suppression of the production of other IL-1 family of pro-inflammatory cytokines^36^. Transgenic mice expressing human IL-37 exhibited an anti-inflammatory function by directly inhibiting the production of pro-inflammatory cytokines^37^. IL-37 was also shown as a key modulator of intestinal inflammation by decreasing IL-1β and TNFα^38^. In addition, IL-37 effectively inhibits the activation of dendritic cells^35^. As IL-37 is involved in anti-inflammation, reduced expression of IL-37 associated with SNP rs3811047 as found in this study (Fig. 1) may cause inflammation, increasing risk of atherosclerosis and CAD.

In conclusion, through a case control association study in two independent populations with a total of 2,501 cases and 3,116 controls, we found significant association between SNP rs3811047 in the *IL37* gene and CAD. We also demonstrated that the minor allele of SNP rs3811047 was associated with a decreased expression level of *IL-37.* Our results suggest that *IL37* is a new susceptibility gene for CAD and that SNP rs3811047 in *IL37* is a new genetic risk factor of CAD.

## Methods

### Study populations

The study subjects were selected from the GeneID database, which is a large ongoing database with clinical data and DNA samples from more than 80,000 Chinese patients and controls. The major goal of GeneID database is to identify susceptibility genes for various cardiovascular diseases in the Chinese Han population^7^,^29^,^39^,^40^.

The diagnosis of CAD was based on coronary angiography, and followed the standard guidelines by the ACC/AHA^41^. The diagnosis was made by more than two independent expert cardiologists. We classified patients with >70% of luminal stenosis in at least one main vessel by coronary angiography, coronary artery bypass graft, percutaneous coronary intervention, and/or a myocardial infarction (MI) as CAD cases^41^. The diagnosis of MI was based on typical chest pain of ≧30 min duration, characteristic electrocardiographic patterns of acute MI, and significant elevation of cardiac enzymes (creatine kinase-MB, lactate dehydrogenase) and troponin I or T^41^. We excluded patients with myocardial spasma and myocardial bridge identified by angiography and those subjects with congenital heart disease, childhood hypertension, and type I diabetes mellitus.

The study included two independent populations. To avoid geographical confounding, we selected one population with study subjects recruited from Northern China and another population with study subjects recruited from Central China. The GeneID Northern population was enrolled from the Northern area of China and had 1,038 CAD cases and 1,076 controls. The GeneID Central population was enrolled from Wuhan in central China and had 1,463 CAD cases and 2,040 controls. In total, our study population included 2,501 CAD cases and 3,116 controls. All subjects were reported to be of Chinese Han origin by self-description or self-report.

The basic demographic and clinical charateristics of the subjects, including the age, gender, hypertension, type 2 diabetes (T2D) and lipid profiles, were obtained from medical records. Hypertension was defined as a systolic blood pressure of ≥140 mm Hg or a diastolic blood pressure ≥90 mm Hg. T2D was diagnosed as a fasting plasma glucose concentration ≥126 mg/dL after at least 8 hours of fasting or a 2-hour plasma glucose level of ≥200 mg/dL during an oral glucose tolerance test (OGTT).

This study was approved by the Ethics Committees on human subject research of Huazhong University of Science and Technology and local institutions and conformed to guidelines set forth by the Declaration of Helsinki. Written informed consent was obtained from subjects following instructions approved by the Ethics Committees.

### SNP selection and genotyping

We selected a non-synonymous taqSNP (rs3811047 in *IL-37*) for this study. SNP rs3811047 is located in the second exon and caused a transition of threonine to alanine at the 42th amino acid residue of IL-37.

The human genomic DNA of each study subject was extracted from the peripheral whole blood samples using the Wizard Genomic DNA Purification Kit (Promega Corporation).

We used the Syto 9 fluorescent dye-based high resolution melt (HRM) method on a Rotor-gene 6200 System (Corbett Life Science) to genotype SNP rs3811047 as described by us^16^,^40^,^42^,^43^,^44^,^45^,^46^,^47^. Primers were designed by software Genetool. The fragment flanking rs3811047 was amplified with the forward primer 5′-AGCCCCCTGGAACCAGGC-3′ and the reverse primer 5′-TCAGCCACCCCCATCACC-3′, together with a final concentration of 5 μmol/L Syto 9 fluorescent dye. The polymerase chain reaction (PCR) was performed with a reaction of a total volume of 25 μL containing 2.5 μL of 10 × PCR buffer, 1.5 mmol/L MgCl_2_, 5 mmol/L dNTPs, 5 pmol of each primer, 25 ng of genomic DNA, 1 μL of Syto 9 fluorescent dye and 1 U of Taq DNA polymerase. The PCR profile was 5 min at 94 °C, 39 cycles of 94 °C for 10 s, 63 °C for 10 s and at 72 °C for 10 s, and a final elongation step at 72 °C for 10 min. Four positive controls were included in each run. The HRM genotyping was verified by direct Sanger sequence analysis of 52 randomly selected samples.

### Quantitative RT-PCR analysis

We performed quantitative RT-PCR analysis to evaluate whether SNP rs3811047 was associated with the mRNA expression level of *IL-37*. The *ΔΔ*Cq method was used to determine the difference of the mean expression levels of *IL-37* among study subjects with different genotypes for rs3811047^16^,^29^,^46^. Quantitative real-time PCR analysis was carried out according to the MIQE guidelines^48^. Total RNA samples were extracted from human peripheral blood leukocytes using Trizol reagent (Life Technologies, Gaithersburg, MD). Quantification of RNA samples was performed using a spectrophotometer (NanoDrop, Thermo Scientific, Hudson, NH). One μg of total RNA was used for reverse transcription with Superscript II reverse transcriptase (Life Technologies, Gaithersburg, MD) and oligo (dT)_18_. A standard two step real-time PCR assay was performed using an ABI 7900-HT Genetic Analyzer (Applied Biosystems, Gaithersburg, MD). Each PCR reaction was performed in a final volume of 10 μL reaction mixture containing 5 μL of 2X PCR master mixture with ROX (Faststart Universal SYBR Green Master Kit, Roche Applied Science, Indianapolis, IN), 2 μL of cDNA, 0.4 μL of 10 pM primers, and 2.6 μL of ddH_2_O. Each reaction was performed in triplicate. The cycling conditions were 95 °C for 10 minutes and 40 cycles of 95 °C for 15 seconds and 60 °C elongation for 45 seconds. After the PCR reaction, Cq values (threshold cycle) of a target gene (*IL-37*) (Cq T) or reference gene *GAPDH (Cq* E) were computed using the RQ Manager program (version 1.3) and SDS (version 2.3). Reaction with a Cq of ≥40 or with the difference between Cq and mean Cq greater than 0.5 were excluded for further analysis. For each individual, the relative expression level △Cq (Cq T-Cq E) of a target gene was normalized with the reference gene and then transformed into relative quantity using RQ formula (RQ = 2^−△△Cq^, ΔΔCq = individual’s ΔCq-calibrator’s ΔCq)^46^. The calibrator was a mixed cDNA sample pooled from 10 randomly selected individuals. The RQ value for the calibrator was normalized to 1. After outliers were excluded, linear regression was used to compare the differences for mean RQ values of *IL-37* between different genotypes of SNP rs3811047.

The sequences for qRT-PCR primers are 5′-AGCTGAAGAAGGAGAAACT-3′ (forward primer) and 5′-CGCCGACTCCAGCATGTTC-3′ (reverse primer) for *IL-37* and 5′-AAGGTGAAGGTCGGAGTCAAC-3′ (forward primer) and 5′- GGGGTCATTGATGGCAACAATA -3′ (reverse primer) for *GAPDH*.

### Statistical analysis

We used PS software 3.0.12 to calculate the statistical power and sample sizes for the case-control design (http://biostat.mc.vanderbilt.edu/wiki/Main/PowerSampleSize). The statistical power of a case control study can be calculated with special parameters, including the minor allele frequency (0.209 for rs3811047 in our study), OR, the numbers of cases and controls, and the Type I error of 0.05. The null hypothesis can be rejected if the odds ratio equals 1 with probability (power). The program uses an uncorrected chi-squared statistic method to evaluate the null hypothesis (http://biostat.mc.vanderbilt.edu/wiki/Main/PowerSampleSize).

The Hardy-Weinberg linkage disequilibrium test among control groups was performed using PLINK 1.06.

For association analysis, χ^2^ tests were performed using Pearson’s 2 × 2 and 2 × 3 contingency tables to calculate the *P* values and corresponding odds ratios (OR) with 95% confidential intervals (CI) by PLINK 1.06 as described by us^7^,^49^,^50^,^51^. Multivariate logistic regression analysis was performed to adjust for some risk factors (age, gender, hypertension, diabetes mellitus and lipid concentrations) using SPSS version 17.0. We used a student’s t-test to compare the continuous variables between cases and controls. Linear regression was used to assess the association between gene expression levels and SNP genotypes. A *P* value of 0.05 or less was considered to be statistically significant.

## Additional Information

**How to cite this article**: Yin, D. *et al*. Genomic Variant in *IL-37* Confers A Significant Risk of Coronary Artery Disease. *Sci. Rep.* **7**, 42175; doi: 10.1038/srep42175 (2017).

**Publisher's note:** Springer Nature remains neutral with regard to jurisdictional claims in published maps and institutional affiliations.

## Figures and Tables

**Table 1:** Demographic and clinical characteristics of study populations.

Characteristics	GeneID Northern Population		GeneID Central Population		GeneID Combined Population		Sex- and Age-Matched Population	
	CAD	Control	CAD	Control	CAD	Control	CAD	Control
N	1,038	1,076	1,463	2,040	2,501	3,116	1,596	1,596
Age (years)	62.01 ± 12.70***	50.52 ± 16.71	64.21 ± 12.44***	49.02 ± 13.88	63.30 ± 13.25***	49.54 ± 15.58	60.05 ± 11.35	60.05 ± 11.35
Male N (%)	680 (65.51)	746 (69.33)	954 (65.21)**	1260 (61.76)	1,634 (65.33)	2,006 (64.38)	1,049 (65.73)	1,049 (65.73)
Hypertension N (%)	594 (57.23)***	202 (18.77)	1007(68.83)***	187 (9.17)	1601 (64.01)***	389 (12.48)	853 (53.45)	208 (13.03)
Diabetes N (%)	145 (13.99)***	84 (7.80)	362 (24.74)***	74 (3.60)	507 (20.27)***	158 (5.07)	316 (19.80)	108 (6.77)
Tch (mmol/L)	4.40 ± 1.08	4.32 ± 1.16	4.68 ± 0.99**	4.45 ± 1.11	4.56 ± 1.08***	4.40 ± 1.07	4.66 ± 1.12***	4.46 ± 1.20
TG (mmol/L)	1.62 ± 1.11***	1.44 ± 1.07	1.59 ± 1.14***	1.46 ± 1.08	1.60 ± 1.12***	1.45 ± 1.10	1.57 ± 1.18***	1.44 ± 1.11
HDL-c (mmol/L)	1.10 ± 0.38**	1.17 ± 0.50	1.14 ± 0.52**	1.20 ± 0.55	1.12 ± 0.40***	1.19 ± 0.41	1.14 ± 0.45**	1.19 ± 0.40
LDL-C (mmol/L)	2.85 ± 0.81***	2.62 ± 0.91	2.88 ± 0.84***	2.62 ± 0.84	2.87 ± 0.82***	2.62 ± 0.88	2.79 ± 0.76***	2.52 ± 0.81

Data are shown as means ± standard deviation (SD) for quantitative variables and n (%) for binary traits.

^**^P < 0.01 between cases and controls for quantitative variables and percent (%) for qualitative variables.

^***^P < 0.001 between cases and controls for quantitative variables and percent (%) for qualitative variables.

**Table 2:** Genotypic analysis of *IL-37* SNP rs3811047 with CAD in the GeneID Chinese Han population under three genetic models.

Cohort (N, Case/Control)	Genotype			Model	*P*_*obs*_	OR (95% CI)	*P*_*adj*_	OR (95% CI)
		Cases (N)	Controls (N)					
GeneID Central (1,503/2,040)	AA	77	60	Additive	1.31 × 10^−3^	n.a	0.46	1.06 (0.91–1.24)
	AG	402	607	Dominant	0.98	1.00 (0.87–1.16)	0.64	0.98 (0.89–1.07)
	GG	984	1373	Recessive	4.72 × 10^–4^	1.83 (1.30–2.59)	1.23 × 10^−3^	1.45 (1.16–1.83)
GeneID Northern (998/1,076)	AA	46	20	Additive	5.80 × 10^−5^	n.a	0.02	1.27 (1.04–1.55)
	AG	349	310	Dominant	3.49 × 10^−4^	1.39 (1.16–1.66)	0.11	1.10 (0.98–1.24)
	GG	643	746	Recessive	6.72 × 10^−4^	2.45 (1.44–4.17)	5.51 × 10^−3^	1.56 (1.14–2.13)
GeneID Combined (2,501/3,116)	AA	123	80	Additive	9.21 × 10^−6^	n.a	1.28 × 10^−2^	1.16 (1.03–1.30)
	AG	751	917	Dominant	1.97 × 10^−2^	1.14 (1.02–1.28)	0.24	1.04 (0.97–1.11)
	GG	1627	2119	Recessive	2.72 × 10^−6^	1.96 (1. 47–2.61)	9.70 × 10^−6^	1.47 (1.24–1.74)

*P*_*obs*_, *P* value from Chi square tests with 2 × 3 contingency tables without adjustment for covariates;

*P*_*adj*_, *P* value adjusted by covariates of sex, age, hypertension, T2D and lipid concentrations by multiple logistic regression analysis;

OR, odds ratio;

95% CI, 95% confidence interval;

Additive model = AA/AG/GG; Dominant model = AA + AG/GG; Recessive model = AA/AG + GG.

**Table 3:** Significant allelic association of *IL-37* SNP rs3811047 with CAD in the GeneID Chinese Han population.

Population	N Case/Control	Frequency of Minor Allele A (Case/Control)	*P*_*hwe*_	*P*_*obs*_	OR (95% CI)	*P*_*adj*_	OR (95% CI)
GeneID Combined	2,501/3,116	0.20/0.17	0.12	3.20 × 10^−4^	1.19 (1.08–1.31)	0.01	1.16 (1.03–1.30)

*P*_*hwe*_, *P* value from Hardy-Weinberg equilibrium tests;

*P*_*obs*_, *P* value from Chi square tests with 2 × 2 contingency tables without adjustment for covariates;

*P*_*adj*_, *P* value adjusted by covariates of sex, age, hypertension, T2D and lipid concentrations by multiple logistic regression analysis;

OR, odds ratio;

95% CI, 95% confidence interval.

**Table 4:** Genotypic analysis of *IL-37* SNP rs3811047 with CAD in age- and sex-matched case control populations under three genetic models.

Population (N, Case/Control)	Genotype			Model	*P*_*obs*_	OR (95% CI)	*P*_*adj*_	OR (95% CI)
		Cases	Controls					
Sex- and age-matched population (1,596/1,596)	AA	113	42	Additive	3.66 × 10^−8^	n.a	1.02 × 10^−3^	1.45 (1.15–1.81)
	AG	471	487	Dominant	0.06	1.16 (0.99–1.34)	0.17	1.10 (0.96–1.29)
	GG	1012	1067	Recessive	4.57 × 10^−9^	2.82 (1.96–4.05)	2.23 × 10^−4^	1.99 (1.45–3.02)

*P*_*obs*_, *P* value from Chi square tests with 2 × 3 contingency tables without adjustment for covariates;

*P*_*adj*_, *P* value adjusted by covariates of sex, age, hypertension, T2D and lipid concentrations by multiple logistic regression analysis;

OR, odds ratio;

95% CI, 95% confidence interval;

Additive model = AA/AG/GG; Dominant model = AA + AG/GG; Recessive model = AA/AG + GG.

**Table 5:** Significant allelic association of *IL-37* SNP rs3811047 with CAD in an age- and sex-matched case control population.

Population	N Case/Control	Frequency of Minor Allele A (Case/Control)	*P*_*hwe*_	*P*_*obs*_	OR (95% CI)	*P*_*adj*_	OR (95% CI)
Age- and sex-matched population	1,596/1,596	0.22/0.18	0.86	1.60 × 10^−5^	1.31 (1.16–1.48)	6.43 × 10^−3^	1.26 (1.08–1.40)
Males	1,049/1,049	0.22/0.18	0.81	1.31 × 10^−3^	1.30 (1.12–1.52)	0.01	1.24 (1.05–1.43)
Females	547/547	0.22/0.18	0.80	8.01 × 10^−3^	1.33 (1.08–1.64)	0.02	1.28 (1.06–1.45)

*P*_*hwe*_, *P* value from Hardy-Weinberg equilibrium tests;

*P*_*obs*_, *P* value from Chi square tests with 2 × 2 contingency tables without adjustment for covariates;

*P*_*adj*_, *P* value adjusted by covariates of sex, age, hypertension, T2D and lipid concentrations by multiple logistic regression analysis;

OR, odds ratio;

95% CI, 95% confidence interval.
